# Characterization of monocyte/macrophage subsets in the skin and peripheral blood derived from patients with systemic sclerosis

**DOI:** 10.1186/ar3066

**Published:** 2010-07-05

**Authors:** Nobuyo Higashi-Kuwata, Masatoshi Jinnin, Takamitsu Makino, Satoshi Fukushima, Yuji Inoue, Faith C Muchemwa, Yuji Yonemura, Yoshihiro Komohara, Motohiro Takeya, Hiroaki Mitsuya, Hironobu Ihn

**Affiliations:** 1 Department of Dermatology & Plastic and Reconstructive Surgery, Faculty of Life Sciences, Kumamoto University, 1-1-1 Honjo, Kumamoto 860-8556, Japan; 2 Department of Blood Transfusion Medicine and Cell Therapy, Faculty of Life Sciences, Kumamoto University, 1-1-1 Honjo, Kumamoto 860-8556, Japan; 3 Department of Cell Pathology, Faculty of Life Sciences, Kumamoto University, 1-1-1 Honjo, Kumamoto 860-8556, Japan; 4 Department of Hematology and Infectious Diseases, Faculty of Life Sciences, Kumamoto University, 1-1-1 Honjo, Kumamoto 860-8556, Japan; 5 The experimental Retrovirology Section, HIV and AIDS Malignancy Branch, National Cancer Institute, 9000 Rockville Pike, Bethesda, MD, 20892, USA

## Abstract

**Introduction:**

Recent accumulating evidence indicates a crucial involvement of macrophage lineage in the pathogenesis of systemic sclerosis (SSc). To analyze the assembly of the monocyte/macrophage population, we evaluated the expression of CD163 and CD204 and various activated macrophage markers, in the inflammatory cells of the skin and in the peripheral blood mononuclear cells (PBMCs) derived from patients with SSc.

**Methods:**

Skin biopsy specimens from 6 healthy controls and 10 SSc patients (7 limited cutaneous SSc and 3 diffuse cutaneous SSc) were analyzed by immunohistochemistry using monoclonal antibody against CD68 (pan-macrophage marker), CD163 and CD204. Surface and/or intracellular protein expression of CD14 (marker for monocyte lineage), CD163 and CD204 was analysed by flow cytometry in PBMCs from 16 healthy controls and 41 SSc patients (26 limited cutaneous SSc and 15 diffuse cutaneous SSc). Statistical analysis was carried out using Mann-Whitney U test for comparison of means.

**Results:**

In the skin from SSc patients, the number of CD163^+ ^cells or CD204^+ ^cells between the collagen fibers was significantly larger than that in healthy controls. Flow cytometry showed that the population of CD14^+ ^cells was significantly greater in PBMCs from SSc patients than that in healthy controls. Further analysis of CD14^+ ^cells in SSc patients revealed higher expression of CD163 and the presence of two unique peaks in the CD204 histogram. Additionally, we found that the CD163^+ ^cells belong to CD14^bright^CD204^+ ^population.

**Conclusions:**

This is the first report indicating CD163^+ ^or CD204^+ ^activated macrophages may be one of the potential fibrogenic regulators in the SSc skin. Furthermore, this study suggests a portion of PBMCs in SSc patients abnormally differentiates into CD14^bright^CD163^+^CD204^+ ^subset. The subset specific to SSc may play an important role in the pathogenesis of this disease, as the source of CD163^+ ^or CD204^+ ^macrophages in the skin.

## Introduction

Systemic sclerosis (SSc) is a multiorgan disease of unknown etiology that is characterized by activation of immune cells, production of autoantibodies and microvascular injury, leading to fibrosis [1-6]. Histopathological hallmarks of SSc are inflammatory infiltrates in early disease stages and accumulation of extracellular matrix proteins resulting in tissue fibrosis. Inflammatory infiltrates are dominated by macrophages and T cells [7,8].

Monocytes, which leave from the bone marrow and enter the circulation, are already mature cells (e.g., phagocytosing microbes and secreting cytokines), but these functions are potentiated by further differentiation into macrophages or dendritic cells in peripheral tissues [9]. Although the definition of the activated macrophages is still controversial, heterogeneity of macrophages has been discussed with regard to different responses to various microenvironmental stimuli. Macrophages are classically activated toward M1 phenotype by microbial products or interferon (IFN)-γ. M1 macrophages have the IL-12^high^, IL-23^high^, IL-10^low ^phenotype and produce nitrogen intermediates and inflammatory cytokines such as IL-1β, TNF-α, and IL-6 to promote active inflammation [10,11]. In contrast, macrophages can be alternatively activated toward M2 phenotype by stimulation with IL-4 or IL-13 [10-12]. They are associated with a high degree of vascularization and wound repair. Also, these macrophages can play a role in certain fibrotic diseases by producing transforming growth factor (TGF)-β [13]. CD204 is considered as one of M2 markers [14,15], whereas CD163 is also known as one of the markers for activated macrophage; CD163 (haemoglobin scavenger receptor) are reported to be up-regulated in active macrophages under IL-10 stimulation [16].

On the other hand, several investigators have reported the increased expression levels of IL-4, IL-13, and IL-10 in SSc serum [17-19]. They are cytokines that are responsible for activation of macrophages as described above, suggesting possible involvement of CD163^+ ^or CD204^+ ^activated macrophages in the pathogenesis of SSc. Macrophages have been thought to be particularly activated in patients with skin disease including SSc and are potentially important sources for fibrosis-inducing cytokines, such as TGF-β [7,8]. In addition, activated circulating monocytes have also been reported in SSc patients, supporting a possible involvement of these cells in the pathogenesis of this disease [20,21]. However, no link between CD163^+ ^or CD204^+ ^monocyte/macrophage lineage and SSc has been established in the skin or in the peripheral blood of SSc patients.

Therefore, to more fully explore the nature of altered immune cell regulation in SSc patients, we showed the distribution of cells with CD163 or CD204 in the skin from healthy controls and SSc patients by immunohistochemistry. In addition, we evaluated the population of CD163^+ ^or CD204^+ ^cells in circulating monocytes of SSc patients by flow cytometric analysis. We suggest that CD163^+ ^or CD204^+ ^macrophages in the skin and the CD14^bright^CD163^+^CD204^+ ^monocyte subset in the peripheral circulating blood are involved in the pathogenesis of SSc.

## Materials and methods

### Patients and controls

Fifty-one SSc patients with a mean (± standard deviation (SD)) age of 57.3 ± 14.1 years were from south Japan and visited the outpatient clinic of the Department of Dermatology, Kumamoto University Hospital, Kumamoto, Japan. Patients were grouped into either diffuse cutaneous SSc (dcSSc; n = 18) and limited cutaneous SSc (lcSSc; n = 33) according to the classification system proposed by LeRoy and colleagues [22]. Table 1 summarizes the patient's clinical features. Skin biopsy specimens and blood samples were obtained from patients after approval by the ethical committee of the Kumamoto University and gaining written informed consent. As healthy controls, 22 disease-free volunteers (mean age; 44.1 ± 42.0 years) were enrolled in the study.

**Table 1:** Clinical features of the study subjects

Characteristics		SSc (n = 51)		Controls (n = 22)
			
		lcSSc(n = 33)	dcSSc (n = 18)	
		47/4		
			
Sex (female/male)		32/1	15/3	18/4

		57.3 (14.1)		
			
Age (years), mean (SD)		59.3 (14.1)	55.1 (12)	44.1 (42.0)

Duration of disease		72.5 (1-240)		
			
month, range (min-max)		74.5 (6-240)	54.5 (1-144)	

Organ involvement (No)	Esophagus	15	3	
	Heart	2	1	
	Kidney	0	0	
	Pulmonary hypertension	0	0	
	Pulmonary fibrosis	0	0	
	Sjögren syndrome	5	9	

ANA Specificity (No)	Anti-topo I	11	13	
	Anti-ACA	10	14	
	Anti-U1 RNP	3	3	

Mean skin score (SD)		2.8 (3.4)	11.9 (9.4)	
Steroid treatment		4	5	

ANA, antinuclear antibodies; Anti-topo I, anti-topoisomerase I antibody; Anti-ACA, anti-centromere antibody; Anti-U1 RNP, anti-uridine 1 ribonucleoprotein antibody; dcSSc, diffuse cutaneous systemic sclerosis; lcSSC, limited cutaneous systemic sclerosis; SD, standard deviation; SSC, systemic sclerosis.

### Antibodies for immunohistochemistry

The following antibodies were used: anti-human-CD68 (dilution 1: 300; mouse anti-human immunoglobulin (Ig) G1, clone KP1; DakoCytomation, Carpinteria, CA, USA), anti-human-CD163 (dilution 1:300; mouse anti-human IgG1, clone 10D6; Novocastra, UK), and anti-human-CD204 (mouse anti-human IgG1, clone SRA-E5, Trans Genic, Kumamoto, Japan) [23,24].

### Immunohistochemistry

Skin biopsy samples from the affected forearms of seven lcSSc and three dcSSc patients were fixed in 10% neutral-buffered formalin, embedded in paraffin, and sliced to a size of 4 μm. After sections were deparaffinized in xylen and rehydrated in a graded ethanol series, antigens were retrieved by incubation with trypsin (Invitrogen, Carlsbad, CA, USA) for 30 minutes for anti-human CD68 antibody. For staining with an anti-human CD163 or CD204 antibody, deparaffinized sections were retrieved by incubation with target retrieval solution (Dako, Carpinteria, CA, USA) or citrate buffer pH 6 for 5 minutes with a microwave oven, respectively. Endogenous peroxide activity was inhibited, after which sections were incubated with 5% normal goat serum for 20 minutes and then reacted with the monoclonal antibodies (anti-CD68, CD163, or CD204 antibody) for six hours at 4°C. After excess antibody was washed out with PBS, samples were incubated with horseradish peroxidase-labelled goat anti-mouse antibody (Nichirei, Tokyo, Japan) for 60 minutes. The reaction was visualized using the diaminobenzidine substrate system (Dojin, Kumamoto, Japan). Slides were lightly counterstained with Mayer's hematoxylin, and examined under a light microscope (OLYMPUS BX50, Tokyo, Japan). Positive cells were counted three times, and results were expressed as the average number of positive cells per 10,000 μm^2 ^using ImageJ (National Institutes of Health, Bethesda, MD, USA).

### Antibodies for flow cytometry

Monoclonal mouse anti-human CD14-phycoerythrin-cyanin 5.1 (PC5) (Beckman Coulter Inc, Fullerton, CA, USA) and anti-human CD163-phycoerythrin (PE) (BioLegend Inc, San Diego, CA, USA) were used for surface immunostaining. Anti-human CD204 (mouse anti-human IgG1, clone SRA-E5) was purchased from Trans Genic (Kumamoto, Japan) and purified using IgG Purification Kit-A (Dojindo Laboratories, Kumamoto, Japan). The purified CD204 antibody was sequentially conjugated with fluorescein isothiocyanate (FITC) using Fluorescein Labelling Kit-NH2 (Dojindo Laboratories, Kumamoto, Japan). The fluorescein/protein ratio was determined by the absorbance of the protein solution at 208 nm and 500 nm. The CD204-FITC antibody was used for both surface and intracellular staining. Appropriately matched isotype control mAb to each antigen-specific monoclonal antibody was used for control.

### Isolation of PBMCs

Peripheral blood mononuclear cells (PBMCs) were obtained from heparinized venous blood, using gradient centrifugation over Ficoll-Paque Plus (Amersham Biosciences Corp, NJ, USA) according to the manufacturer's protocol.

### Flow cytometry

The cell number of isolated PBMCs (approximately 5 × 10^5^) were determined with Burker-turk line (Erma, Tokyo, Japan) and blocked FcR with FcR Blocking Reagent (Miltenyi Biotec GmbH, Bergisch Gladbach, Germany) for 10 minutes at 4°C. Then, the cells (5 × 10^5 ^) in each test tube was incubated for staining with isotype-matched control antibody or relevant antibody (CD14, CD163 or CD204) for 15 minutes at room temperature in the dark. For intracellular staining, the cells were fixed and permeabilized according to manufacturer's instructions using Intra Prep (Beckman Coulter Inc, Fullerton, CA, USA). Then cells were washed once in PBS and further incubated for intracellular staining with isotype-matched control antibody or relevant antibody CD204 for 15 minutes at room temperature in the dark. Finally, cells were washed three times, resuspended in 500 μl of PBS containing 0.5% formaldehyde, and analyzed using a Cytomics FC 500 (Beckman Coulter Inc, Fullerton, CA, USA). The data was converted with FlowJo software (Tree Star Inc, Ashland, OR, USA). Monocytes were gated based on forward-sideward scatter profiles and CD14 expression was used to verify whether the gated cells were indeed monocytes.

### Statistical analysis and clinical significance

Statistical analysis was carried out using Mann-Whitney U test for comparison of means and Spearman's correlation coefficient by rank for evaluating correlation of factors. Each protein expression level detected by flow cytometry was represented by mean fluorescence intensity (MFI). *P *values less than 0.05 were considered statistically significant. All data were expressed as mean ± SD.

## Results

**Figure 1:**
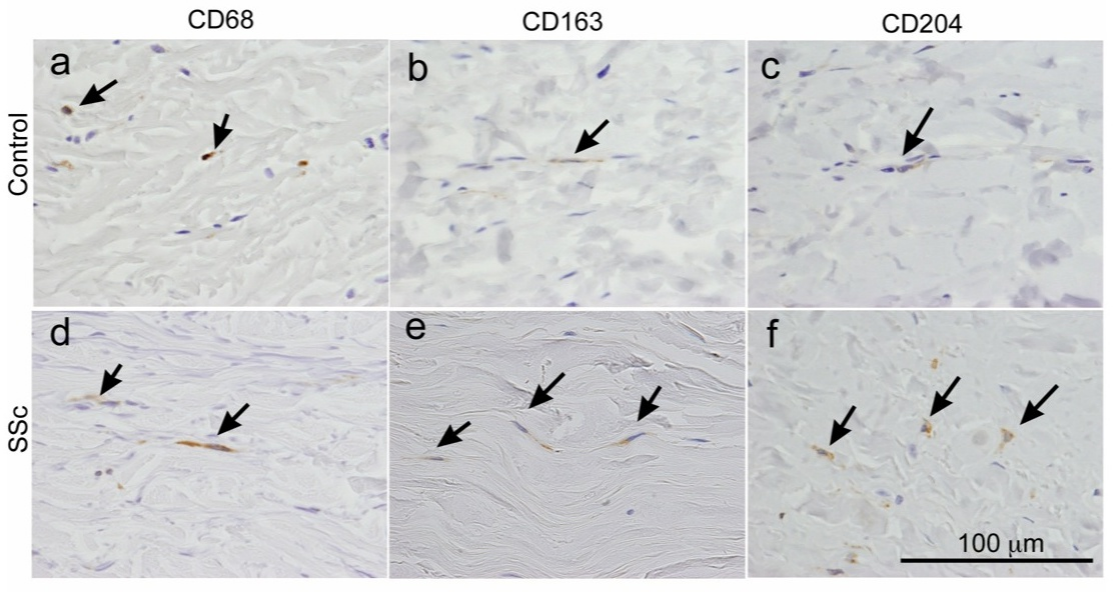
**Immunoperoxidase staining of skin in SSc patients compared with that in healthy controls**. Skin sections from **(a to c) **healthy controls and **(d to f) **systemic sclerosis (SSc) patients were stained with anti-human-CD68, CD163 and CD204 antibody, respectively. A larger number of **(d) **CD68^+ ^cells, **(e) **CD163^+ ^cells and **(f) **CD204^+ ^cells in SSc skin were distributed not only in the perivascular and periappendageal regions, but also between thickened collagen fibers compared with that in healthy control skin (a, b and c, respectively). Arrows indicate positively stained cells (brown); nuclei are counterstained with hematoxylin. Results are representative of 6 controls and 10 SSc patients. Bar = 100 μm.

First, we determined whether activated macrophages are infiltrated in SSc skin. We found cells positive for CD68 (pan-macrophage marker), CD163 and CD204 were increased not only in the perivascular regions but also between thickened collagen bundles in the skin of SSc patients (Figure 1). When the cell number in randomly selected areas (per 10,000 μm^2^) was counted, the number of CD68^+^, CD163^+ ^or CD204^+^cells in the skin of SSc patients was larger than that of healthy controls (5.3 ± 2.5 vs 2.2 ± 0.2, 4.0 ± 2.0 vs 1.3 ± 1.0 and 3.5 ± 1.6 vs 1.5 ± 0.5, respectively; *P *< 0.05). In addition, to evaluate a contribution of macrophages to fibrosis, we further counted the number of macrophages between collagen bundles, excluding perivascular and periappendageal infiltrated macrophages (Table 2). There was no difference in the number of CD68^+ ^cells between healthy controls and SSc patients. However, the number of CD163^+ ^cells and CD204^+ ^cells between the collagen fibers in the skin of SSc patients were significantly greater than that of healthy controls (3.8 ± 0.8 vs 1.2 ± 0.5 and 3.6 ± 0.6 vs 0.4 ± 1.6, respectively; *P *< 0.05, Table 2). The number of CD68^+^, CD163^+ ^or CD204^+ ^cells in the randomly selected areas or between collagen bundles of dcSSc skin did not significantly differ from that of lcSSc skin. Thus, macrophages between collagen fibers were activated to express CD163^+ ^and CD204^+ ^in SSc skin, without changing the total number of macrophages.

**Table 2:** Results of immunohistochemical staining

Number of positive cells (10000 μm^2^)	SSc (n = 10)		Controls (n = 6)	*P *value
			
	lcSSc (n = 7)	dcSSc (n = 3)		
	3.2 ± 0.7			> 0.05
			
CD68	2.9 ± 1.0	3.2 ± 0.4	2.0 ± 0.2	> 0.05

	3.8 ± 0.8			< 0.05*
			
CD163	4.0 ± 1.1	3.6 ± 0.5	1.2 ± 0.5	< 0.05*

	3.6 ± 0.6			< 0.05*
			
CD204	3.4 ± 0.7	3.8 ± 0.4	1.4 ± 0.4	< 0.05*

Data are expressed as mean ± standard deviation; *P *< 0.05*.

The number of CD163^+ ^and CD204^+ ^cells between collagen fibers was significantly greater in SSc patients compared in healthy controls.

dcSSc, diffuse cutaneous systemic sclerosis; lcSSC, limited cutaneous systemic sclerosis; SSC, systemic sclerosis.

**Figure 2:**
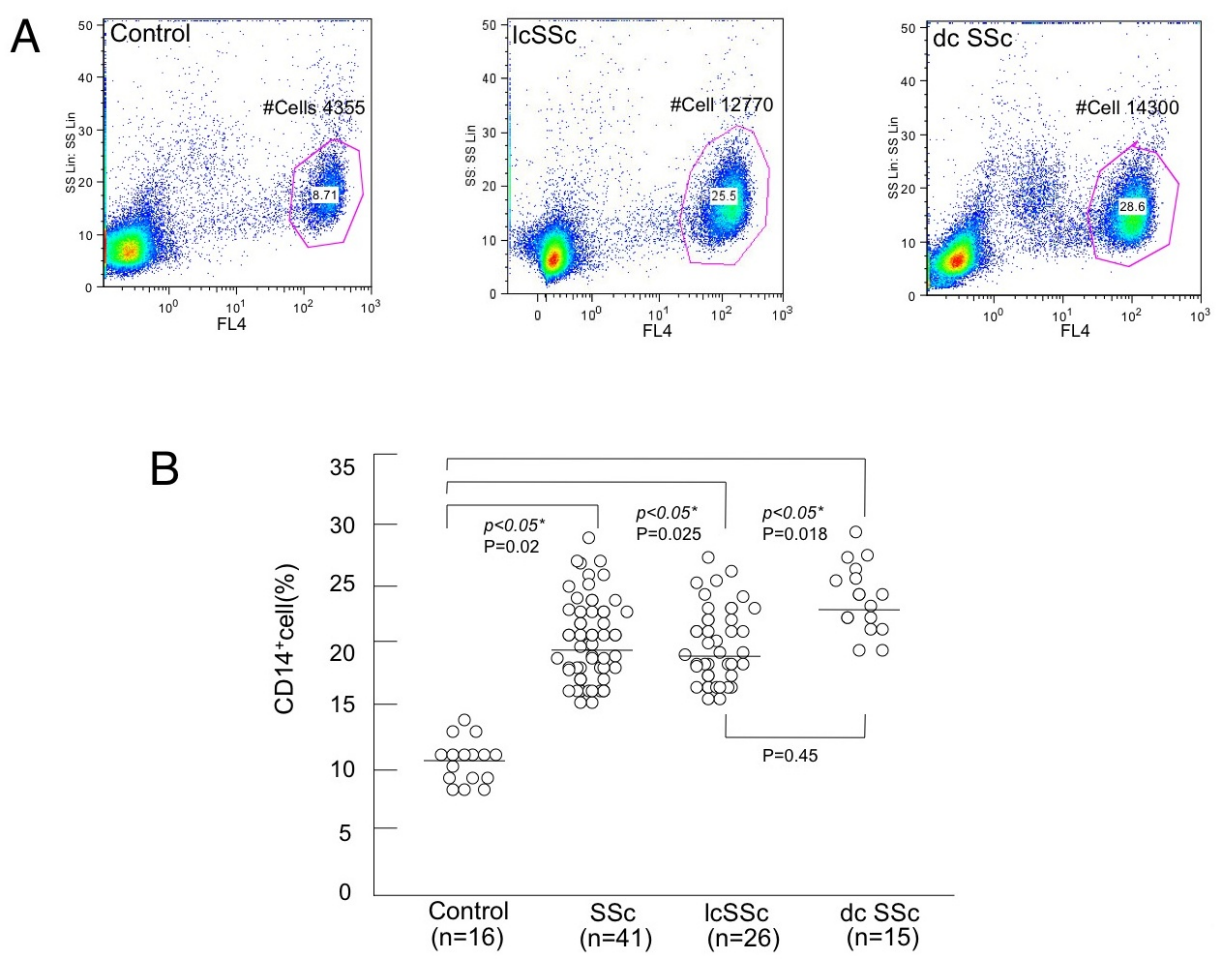
**Increased number of CD14^+ ^cells in (PBMCs) from SSc patients**. Peripheral blood mononuclear cells (PBMCs) isolated from healthy controls or systemic sclerosis (SSc) patients were analyzed by single-color flow cytometry for CD14 expression. Upper panel **(a) **shows the results of PBMCs from healthy controls (left), limited cutaneous systemic sclerosis (lcSSc) patients (middle), and diffuse cutaneous systemic sclerosis (dcSSc) patients (right). Values are the percentage of total PBMCs in each region. FL4, phycoerythrin-cyanin 5.1 fluorescence; SS, side scatter; #Cells = actual number of the cells. Data presented here are representative of 16 healthy controls and 41 SSc patients. Lower panel **(b) **depicts the summary of results, comparing percentages of CD14 positive cells in PBMCs (shown on the ordinate) from healthy controls and SSc patients. *P < 0.05 as compared with the value in cells from healthy controls.

**Figure 3:**
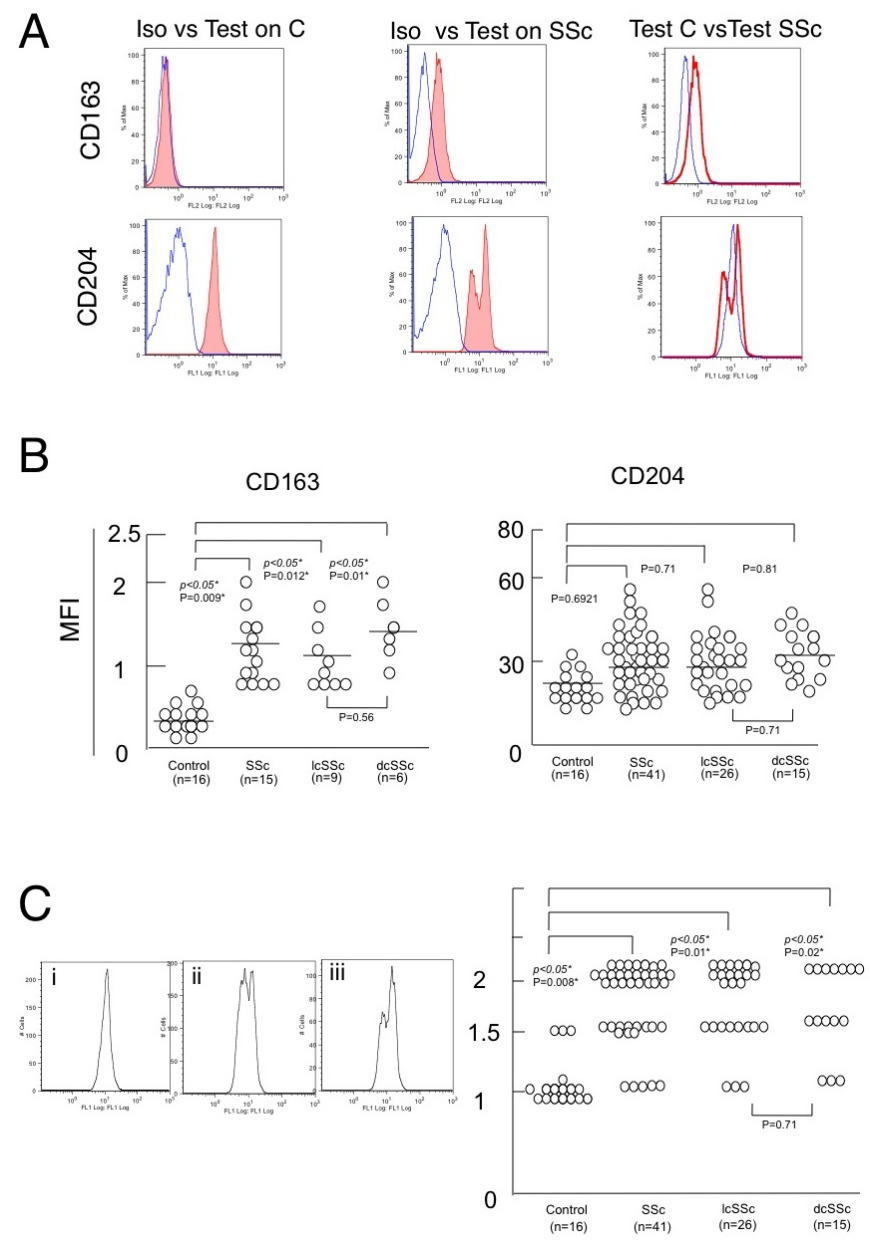
**Expression level of CD163 and CD204 in CD14^+ ^PBMCs**. **(a) **Left panels (healthy controls) and middle panels (systemic sclerosis (SSc) patients) depict staining with each isotype-matched monoclonal antibody control (Iso) (unfilled graph) and antigen-specific monoclonal antibody indicated (Test) (filled graph). Right panels depict staining with each monoclonal antibody in healthy controls (Test C) (fine line) versus SSc patients (Test SSc) (bold line). Data presented here are representative of 16 healthy controls and 15 (for CD163) or 41 (for CD204) SSc patients. **(b) **Comparison of expression level of CD163 and CD204 in CD14^+ ^peripheral blood mononuclear cells (PBMCs) between healthy controls and SSc patients. Mean fluorescence intensity (MFI) are shown on the ordinate. *P < 0.05 as compared with the value in cells from healthy controls. **(c) **The left panel shows the representative pattern of CD204 histogram. The number of peaks was counted as 1 in the **(i) **left panel, **(ii) **1.5 in the middle panel and **(iii) **2 in the right panel. The right panel shows increased number of peaks in CD204 histogram in SSc patients. The number of peaks is shown on the ordinate. * P < 0.05 as compared with the value in cells from healthy controls.

To answer the question why the infiltration of activated macrophage is increased in SSc skin, we then examined PBMCs positive for CD14, which is widely accepted as a useful marker of monocytes lineage, in SSc patients by flow cytometry (Figure 2a). The population of CD14^+ ^PBMCs was significantly greater in SSc patients than that in healthy controls (19.6 ± 7.8% vs 11.5 ± 5.1%, *P *> 0.05; Figure 2b). There was no significant difference between lcSSc and dcSSc patients in the size of the population (*P *= 0.45). We confirmed that the CD14^+ ^populations were monocytes, by profiles of forward and side light scatter. We next investigated expression levels of activated macrophage markers (CD163 and CD204) in CD14^+ ^PBMCs of SSc patients and healthy controls by detecting MFI (Figure 3a). We decided to stain both surface and intracellular CD204 because MFI of each surface or intracellular CD204 was not high enough to detect (data not shown). Additionally, it has been also reported that CD204 are mainly expressed in the endoplasmic reticulum, nuclear envelope and Golgi apparatus of non-monocyte lineage cells but moved to the cell surface and endosomes of monocytes lineage-like cells [24]. MFI of CD163 in CD14^+ ^PMBCs of both lcSSc and dcSSc patients was significantly greater than that of healthy controls (Figures 3a and 3b), suggesting that monocytes in SSc PMBCs already express activated macrophage markers. On the other hand, MFI of CD204 in CD14^+ ^PMBCs of lcSSc or dcSSc patients was not significantly altered compared with those of healthy controls (Figure 3b). Of note, a histogram of surface and intracellular CD204 showed two peaks; apparently weak and strong positive populations (Figures 3a and 3c). Staining only surface CD204 did not show such unique peaks in both healthy controls and SSc patients (data not shown). Indeed, the number of peaks in the CD204 histogram in SSc patients was significantly larger than that in healthy controls (Figure 3c).

**Figure 4:**
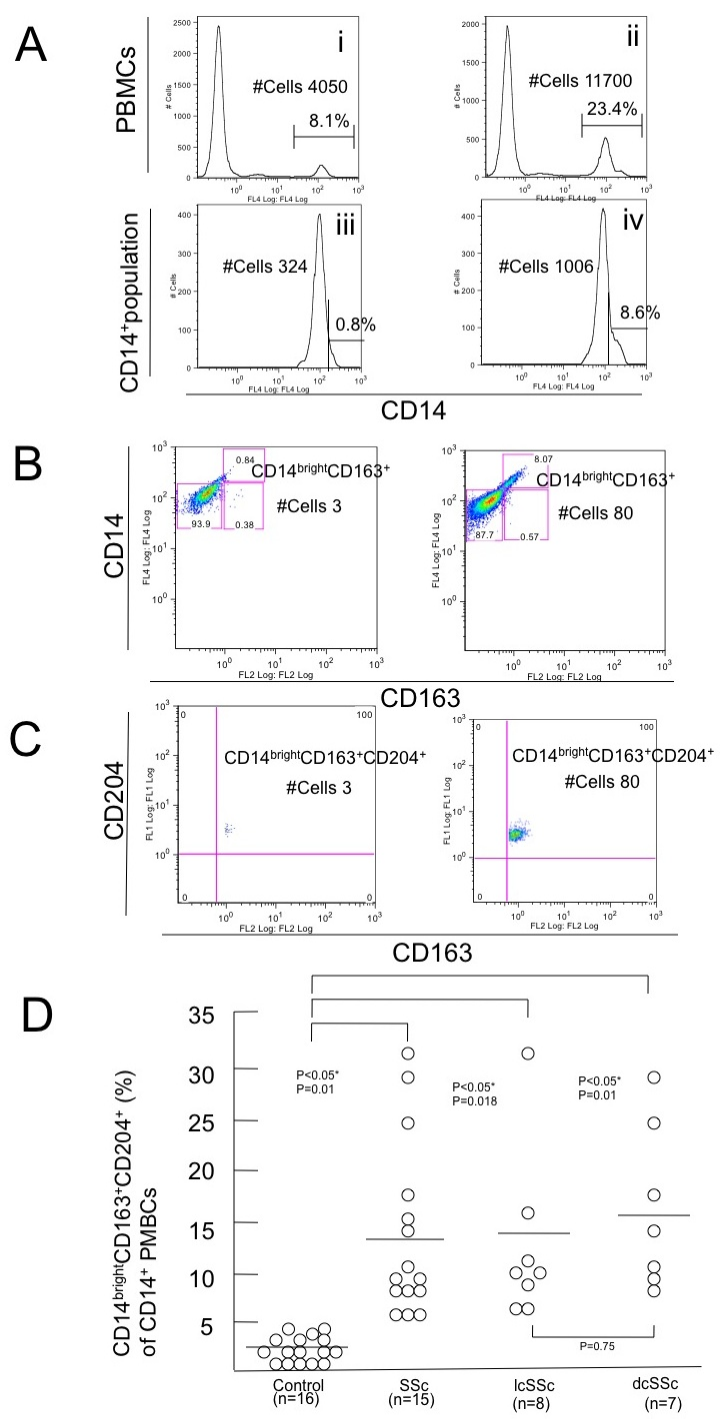
**Gating process for detecting CD14^bright^CD163^+^CD204^+ ^cells in CD14^+^hPBMCs**. **(a) **Histogram representation of CD14^+ ^cells in peripheral blood mononuclear cells (PBMCs) by three-color staining. Upper panels show the percentage of CD14^+ ^cells in PBMCs from **(i) **healthy controls and **(ii) **systemic sclerosis (SSc) patients. Lower panels show enlarged histogram representation of the CD14^+ ^population in PBMCs from **(iii) **healthy controls and **(iv) **SSc patients. Values over the horizontal bar are the percentage of CD14^bright ^cells in CD14^+ ^population. **(b) **Dot plot representation of CD163^+ ^cells in CD14^+ ^population of PBMCs from healthy controls (left panel) and SSc patients (right panel). CD163^+ ^cells belonged to the CD14^bright ^subpopulation, and the percentage of CD14^bright ^CD163^+ ^subpopulation in SSc patients was increased compared with that in healthy controls. **(c) **Dot plot representation of CD163^+ ^and CD204^+ ^cells in CD14^bright^CD163^+ ^population of PBMCs from healthy controls (left panel) and SSc patients (right panel). Further extended dot plot representation of CD14^bright^CD163^+^subpopulation (an upper right quadrant of Figure 4b) revealed that these cells co-express CD204. Data presented in a to c are representative of 16 healthy controls and 15 SSc patients. #Cells = actual number of the cells. **(d) **The graphic representation of quantitative result of CD14^bright^CD163^+^CD204^+^cells in PBMCs of SSc patients and healthy controls. Percentage of CD14^bright^CD163^+^CD204^+ ^cells determined by flow cytometry is shown on the ordinate. *P *< 0.05.

To further characterize the feature of CD14^+ ^PBMCs, we performed three-color staining by CD14-PC-5, CD163-PE, and CD204-FITC antibodies. Increased percentages of CD14^+ ^cells in PBMCs from SSc patients compared with that from healthy controls (Figure 2) was confirmed by three-color staining (23.4% vs 8.1%, Figures 4a i and ii). Extended histogram representations clearly revealed that there exists CD14^dim ^and CD14^bright ^subpopulations in CD14^+^PMBCs and that the percentages of CD14^bright ^subpopulations was increased in SSc patients compared with that from healthy controls (8.6% vs 0.8%; Figures 4a iii and iv). Additionally, the dot plot representation of CD14 and CD163 staining demonstrated that CD163^+ ^cells belong to the CD14^bright ^subpopulation in both healthy controls and SSc patients (Figure 4b). Extended dot plot representation also revealed the CD14^bright^CD163^+^subpopulation co-expressed CD204 (Figure 4c). The population of CD14^bright^CD163^+^CD204^+ ^subset in SSc PBMCs was significantly greater than that in healthy controls (11.0 ± 9.0% vs 1.2 ± 0.1%, *P *< 0.05; Figure 4d). Taken together, the presence of the CD14^bright^CD163^+^CD204^+ ^monocyte subset may be characteristic in SSc PBMCs.

## Discussion

Our study revealed three major findings. First, in the skin from SSc patients, CD163 and CD204-positive activated macrophages between the collagen fibers was significantly increased compared with that in healthy controls. Second, in PBMCs from SSc patients, the population of CD14^+ ^cells was significantly greater than that of healthy controls. Third, the expression levels of CD163 in SSc CD14^+ ^PBMCs was also significantly greater than that in healthy controls and the CD163^+ ^cells belong to the CD14^bright^CD204^+ ^population in SSc patients.

Expression of CD204 has been associated with an anti-inflammatory M2 macrophage phenotype and believed to be useful for distinguishing the M2 macrophages from the pro-inflammatory M1 macrophages [14,15,25]. M2 macrophages are known to be increased in the early stage of fibrosis. These cells release a number of proinflammatory and fibrogenic mediators such as TGF-β [17]. We previously reported an increased number of M2 macrophages, CD204^+ ^cells, in the skin from patients with localized scleroderma [26]. This study suggests an important role of M2 macrophage (i.e. secretion of TGF-β) in the pathogenesis of SSc as well as localized scleroderma.

The exact mechanism or significance of CD163 expression on the active macrophages is unclear and the naming of different macrophage subsets is still evolving, although a recent article described that M2 macrophages can be further subdivided into M2a (after exposure to IL-4 or IL-13), M2b (forming immune complexes in combination with IL-1β or lipopolysaccharide) or M2c (after exposure to IL-10, TGF-β or glucocorticoids) [27]. Macrophages positive for CD204 and CD163 may play a major role in the formation of tissue fibrosis.

The population of CD14^+ ^cells in circulating monocytes in SSc patients has been reported [21]. Andrews and colleagues [21] did not find a significant difference in the percentage of CD14^+ ^PBMCs between SSc and healthy controls using a fluorescence microscopy method, whereas an increased population of CD14^+ ^PBMCs in SSc patients were found by flow cytometry in our study. This discrepancy might be explained by the different method used in the earlier study. Reduced or increased numbers of CD14^+ ^cells under pathological conditions have been reported [28,29]. Circulating CD14^+ ^monocytes originate from hematopoietic stem cells in the bone marrow. It has been recently reported that the addition of IL-10 into freshly isolated human monocytes results in a considerable increase in CD14 mRNA *in vitro *[30]. Increased numbers of CD14^+ ^cells in our study may be partly interpreted as the direct result of increased serum IL-10 in SSc patients, as described above [17,19]. Therefore we hypothesized that increased numbers of circulating CD14^+ ^cell could be a source for the increased number of CD68^+ ^macrophages in the skin of SSc patients, which are derived from migrated monocytes.

The expression levels of CD163 in PBMCs of SSc patients were significantly higher than those of healthy controls in our study. On the other hand, Andrews and colleagues [21] also reported that monocytes in SSc patients differed from those in normal controls and appeared to have undergone advanced differentiation and activation change (i.e. increased number of larger and lightly stained esterase positive cells, decreased lectin peanut agglutinin binding and decreased number of Leu M2 surface antigen-positive cells). The significance of CD163 expression on monocytes (not macrophages) is also unknown. Higher CD163 expression in SSc CD14^+ ^PBMCs in our study may indicate advanced differentiation of monocytes into an active state. Considering that CD163 expression on monocytes are modulated by pro-anti-inflammatory mediators such as IL-10 [16,31], an increased number of CD163^+ ^cells in CD14^+^PBMCs in our study may be interpreted as the direct result of increased serum IL-10 in SSc patients [17-19]. Also, another investigator recently reported that increased gene expression levels of CD204 in PBMCs in patients with acute coronary syndrome and concluded that the CD204 levels could be a predictive marker for a reattack of cardiovascular event [32]. Thus, our finding of the unique pattern of CD204 histogram in SSc CD14^+ ^PBMCs may indicate abnormal activation of monocytes. As CD14^bright^CD163^+^CD204^+ ^cells were not apparent in PBMCs from healthy controls, the presence of this monocyte subset may be highly specific to this disease. Taken together, this subset of monocytes may play an important role in SSc as the source of CD163^+ ^or CD204^+ ^macrophages in the skin.

Our results have some limitations. First, we could not perform triple staining of CD68, CD163 and CD204 on the same macrophage using serial skin sections. Second, we could not find any correlation between the expression of CD163 or CD204 in skin macrophages or the percentage of CD14^bright^CD163^+^CD204^+ ^cells in PBMCs and serum levels of cytokines (e.g. IL-10), the presence of known disease-related factors (e.g. modified Rodnan's skin score) or treatments as far as we determined (data not shown). Further study will be needed in the future.

## Conclusions

In the light of reported evidence, including present results, we conclude that CD163^+ ^or CD204^+ ^activated macrophages may be one of the potential fibrogenic regulators in the SSc skin. Furthermore, this study suggests a portion of PBMCs in SSc patients abnormally differentiates into a CD14^bright^CD163^+^CD204^+ ^subset. Further studies to analyze the involvement of macrophages/monocytes will provide a better understanding of the pathogenesis of this disease.

## Abbreviations

dsSSc: diffuse cutaneous systemic sclerosis; FITC: fluorescein isothiocynate; IFN-α: interferon-α; Ig: immunoglobulin; IL: interleukin; lcSSc: limited cutaneous systemic sclerosis; mAb: monoclonal antibodies; MFI: mean fluorescence intensity; PBMCs: peripheral blood mononuclear cells; PBS: phosphate-buffered saline; PC5: phycoerythrin-cyanin 5.1; PE: phycoerythrin; SD: standard deviation; SSc: systemic sclerosis; TGF-β: transforming growth factor-β; TNF-α: tumour necrosis factor-α.

## Competing interests

The authors declare that they have no competing interests.

## Authors' contributions

HI and MJ designed the study. NHK, MT, YY, and FCM assisted in the study design, oversaw the project running and data analysis, and drafted the manuscript. TM, SF, YI, YK, and HM assisted in the study design and coordination, and oversaw the data analysis and drafting of the manuscript. All authors read and approved the final manuscript.
